# Symptom changes in multiple sclerosis following psychological interventions: a systematic review

**DOI:** 10.1186/s12883-014-0222-z

**Published:** 2014-11-30

**Authors:** Francesco Pagnini, Colin M Bosma, Deborah Phillips, Ellen Langer

**Affiliations:** Department of Psychology, Università Cattolica del Sacro Cuore, Milan, Italy; Niguarda Ca’ Granda Hospital, Milan, Italy; Department of Psychology, Harvard University, Cambridge, MA USA

**Keywords:** Multiple sclerosis, Psychological interventions, Physical health outcomes, Mind/body connection

## Abstract

**Background:**

Multiple Sclerosis is a disease of the central nervous system involving a variety of debilitating physical, sensory, cognitive and emotional symptoms. This literature review evaluated the impact of psychological interventions on the physiological symptoms associated with the illness.

**Methods:**

A systematic literature search was conducted using Medline, PsycINFO, Scopus, and the Cochrane Library databases, as well as reference lists. Relevant studies were selected and assessed according to a preset protocol.

**Results:**

The search produced 220 articles, with 22 meeting inclusion criteria for the review. A total of 5,705 subjects with Multiple Sclerosis were analyzed. Results from the included studies indicate a general improvement in both psychological and physiological outcomes following psychological treatment. The most highly influenced physical symptoms include fatigue, sleep disturbances, pain, and physical vitality.

**Conclusions:**

Findings from the review suggest a positive relationship between psychological interventions and physiological Multiple Sclerosis symptoms. Implications for future research are discussed.

## Background

This paper provides a brief literature review concerning the relationship between physiological difficulties associated with Multiple Sclerosis (MS) and psychological interventions intended to remediate or otherwise improve functionality and quality of life. Our specific focus is the unearthing of evidence that psychological or behavioral treatments have an impact on both the psychological well-being and the physiological consequences of the disease. We are also interested in exploring the relationship between MS symptoms and psychological issues within the broader framework of the mind-body connection. The paper summarizes the structure of this framework along with the trends in the literature; presents the etiology and primary physiological consequences of MS; a summary of its psychological consequences investigated thus far; and an application of the mind/body hypothesis in the MS field. We identify some key gaps in related research and propose potential areas for further work to address these gaps.

### Consciousness and the brain: the mind-body connection

The classic mind-body problem searches for an understanding of the distinctions – or lack thereof – between physical and mental entities: Is the physical brain a distinct entity from its mental processes? How do we know, how is it defined, and what implications are there for how we understand and treat our health?

Conceptions of a *dualistic* framework follow the early work of Descartes who proposed that the mind is a nonphysical substance distinct from the brain, that our mind (and all it encompasses) is different from our physical brain in its fundamental composition as matter. In contrast to that is the argument for *materialism* proposing that because the world consists only of matter, there is no true distinction between the mental and the physical, i.e., that all mental states, properties, and processes are connected and interact with physical states, properties, and processes. Much of Western culture continues to preserve a belief in the dichotomy between mind and body.

We see dualism in medical science, which maintains the notion that disorders stem from either the physical or the mental, treating the mind and the body separately [1]. Although there was an appeal that from the field of psychiatry is now beginning to extend over different medical specialties [2] for a more comprehensive treatment model (i.e., a bio-psycho-social approach), a strictly biomedical approach to physical illnesses persists, treating physical bodily symptoms to the exclusion of mental health [3]. Findings from more current research suggest that is changing in several areas, proposing correlations between consciousness and the brain [4]; that negative emotions (e.g., depression and anxiety) are highly associated with the development of coronary heart disease [5]; and that such emotions have a negative effect on cardiovascular and immune system responses [6]. Further research suggests that negative emotions can produce direct and indirect influence on state and trait pain [7] and fatigue [8]. Similarly, Pressman and Cohen [9] suggest that positive emotions can lead to an improvement in physical health, such as increased physical functioning in adults, protection against infectious illness, and lower mortality rates.

The link between mind and body has been proposed going back as far as the 1970s, when Ellen Langer conducted one of the first tests of the mind/body unity theory on disease and ageing. Langer [10] suggested that a healthy mind would put the body in a healthier place, forming the basis for the 1980 “counterclockwise study”, in which Langer and her students studied what effects of turning back the clock psychologically would have on the physiological states of the participants [11]. The results of this study changed the way we view not only aging (the cohort being elderly men) but also of traditional western notions of “limits” - that biology is not destiny, that “it is not primarily our physical selves that limit us but rather our mindset about our physical limits” [10].

### Physiology and key psychological consequences of multiple sclerosis

Multiple Sclerosis is a chronic degenerative disease of the central nervous system that involves functionality of the brain and spinal cord, with physical, sensory, cognitive and emotional responses ranging from mild to severe. The relatively high variability in symptomology is determined primarily by the location of the lesions in the brain and spinal cord. Lesions in the frontal and parietal lobes result in cognitive and emotional problems; plaque in the cerebrum, brain stem and spinal cord result in functional limitations of extremities [12]. In this sense, MS is a highly individual disease, prompting interventions targeting broad categories of disease progression and psychosocial impacts. The National Multiple Sclerosis Society (NMSS) estimates prevalence of MS in the United States at 400,000, and global prevalence at over 2 million people [13].

A diagnosis of MS often has profound social and psychological consequences. Because MS usually strikes individuals in their most productive years, its impact can be overwhelming [14]. The unpredictable and variable nature of MS also makes it particularly difficult to accept. The newly diagnosed individual is faced first with the shock of a disease, which is chronic and unpredictable in its course, often with progressive impacts on critical spheres of functioning. The future undoubtedly promises reduced physical function and disability, along with disruptions in education, employment, sexual and family functioning, friendships and activities of daily living. The grim prognosis and the added unpredictability of day-to-day health in relapse-remitting MS and side effects of medication greatly impacts upon quality of life [15].

Multiple Sclerosis can also have a considerable influence on the individual’s sense of self [16,17]. Physical changes and functional limitations may lead to a sense of loss of identity or role strain especially when the individual can no longer perform previously valued activities [15]. It is frequently necessary to redefine one’s self-image in order to incorporate the limitations imposed by MS. Each time the individual experiences a new loss of function this sense of loss may be renewed. One of the major sources of psychological distress related to the physical impairments is sexual dysfunction. The most frequent complaints are erectile and ejaculatory dysfunctions in men, vaginal lubrication in women, and a loss of libido and difficulty in achieving orgasm in both genders [18]. This problem covers significant aspects of life and can arise at any time during the course of MS, with a prevalence that varies between 50% and 90% [19].

Depressive features are often reported by people who have MS. Lifetime prevalence of major depressive disorder (MDD) is approximately 50% [20]. This is three times the rate reported in the general population [21]; the high prevalence may have multiple etiologies, including psychosocial factors such as the difficulty to deal with one’s emotions, and lack of social support [22]. Depression is one of the main determinants of quality of life and may further compromise cognitive function, and may lead to suicidal intent. It often impairs relationships and reduces compliance with disease- modifying treatments [23]. In addition, people with MS and MDD have been found to suffer from high levels of anxiety [24].

### Relationship between physiological and psychological features in multiple sclerosis

Depressive features following the onset of MS physiological symptoms may not be a simple psychological reaction to MS, but instead may be related to biological aspects of the illness itself [25]. Biological processes such as inflammation, neuroendocrine dysfunction or regional brain damage [26] are likely to be at least partly responsible for depressive features.

The relationship between psychological issues and MS symptoms has been underestimated in the past, but there is growing evidence of increased interest within the scientific community. For example, a number of prospective studies suggest that psychological stress increases relapse risk in MS [27]. We believe that more focused investigations into the physiological outcomes of a psychological intervention may lead to a better understanding of therapeutic options for people with MS.

It is also possible that the relationship between the underlying biological mechanism of MS and depressive symptoms works in two directions. If that is the case, successful treatment of depression utilizing behavioral approaches could also affect the underlying MS physiology, encouraging consideration of psychological interventions that could reduce the symptomatology of the illness and moving from a palliative care framework for behavioral treatments to a potentially therapeutic one. There is little direct evidence for this hypothesis currently, primarily because behavioral interventions rarely include biological markers or even consider symptoms assessment among the outcomes. Thus far, psychological outcomes are most often the only ones expected and assessed at the end of a behavioral treatment [28], without considering the possibility that an intervention could impact physiological measures.

## Methods

This review focuses on psychological interventions for the treatment of Multiple Sclerosis. A systematic search strategy was conducted with Medline, PsycINFO, Scopus and the Cochrane Library using the search terms “multiple sclerosis” in combination with “psychological intervention”, “psychological treatment”, “psychotherapy” or “psychological therapy”. Further articles were included from the reference lists of review articles. There were no time limitations for the bibliographic search, but emphasis was placed on recent publications, post 2004. The list of articles provided by database and article reference search were screened for articles that investigate the effects of psychological interventions on physical health and symptoms of people with MS. Two reviewers independently assessed articles. Susceptibility to bias was evaluated following the QUOROM Statements [29]. Only articles published in English from peer-reviewed journals were considered. Four criteria were used to select studies: 1) the study reported either primary or secondary outcomes on physical health, either self-reported or instrumentally assessed; 2) the study investigated the effects of a psychological intervention; 3) the outcomes resulted from a comparison between groups, with a randomized controlled trial; 4) study results referred to a minimum of 10 participants. Data from the included article were extracted and reported into an Excel spreadsheet. The review includes randomized clinical trials, that intrinsically present a risk of bias. It is therefore expected that results will provide a reliable recommendation (Evidence Level 1) [30].

## Results

Database and article references search provided a list of 220 papers. Twenty-two articles met the criteria for the inclusion in the review. Included studies and their properties are reported in Table 1.

**Table 1:** **Included studies**

**Study**	**Patients (n)**	**Severity of symptoms**	**Mean disease duration**	**Mean age**	**Type of intervention**	**Duration of the intervention**	**Type of control**	**Results on psychological variables**	**Results on symptoms**
Barlow et al. [31]	216	N/R	12 years		Chronic Disease Self-Management Course, a lay-led self-management intervention that provides participants with a range of skills and strategies	6 weeks	Waiting-list	CDSMC had an impact on self-management self- efficacy and trends towards improvement on depression and MS self-efficacy were noted. All improvements were maintained at 12-months	CDSMC had an impact on MSIS physical status
Stuifbergen et al.[32]	113	15.65 on the Incapacity Status Scale	10.76 years	45,79	lifestyle-change classes and telephone follow-up	8 weeks	Waiting-list	Improvement of self-efficacy, health-promoting behaviors and mental health (SF36)	Reduction of Bodily Pain as measured with the SF36, no difference on the severity of impairment as measured with the Incapacity Status Scale
Ghafari et al. [33]	66	EDSS <5.5	2 years	31,5	Progressive Muscle Relaxation Technique	63 sessions during two months	No intervention	One and two months after intervention the experimental group reported better QoL	The physical component of QoL (PCS-8) improved as well
Tesar et al. [34]	29	EDSS <5.5 (mean 3.2)	5.1 years	38.2	Psychological program which combines proven cognitive-behavioral strategies for coping with stress with body exercises	7 weeks	Waiting-list	The therapy group showed long-term improvements in depressive stress coping style	The therapy group showed short-term improvement in “vitality and body dynamics”.
Forman & Lincon [35]	40	23 on the Guys Neurological Disability Scale	9.8 years	47.5	The intervention group programme was designed for people with multiple sclerosis and focused on adjustment to illness.	6 weeks	Waiting-list	Patients allocated to the group intervention reported fewer depressive symptoms than those in the control group but there were no significant differences in anxiety symptoms, self-efficacy or quality of life.	No changes on the MS Impact Scale - Physical
O’Hara et al. [36]	183	17 (median) on the Barthel Index	11.8 years	51.5	The intervention comprised discussion of self-care based on client priorities, using an information booklet about self-care.	The discussions lasted between 1 and 2 hours and were conducted on two occasions, over a one month period.	No intervention	At follow-up the intervention group had better SF-36 health scores, in mental health and vitality. Participants in the intervention group had maintained levels of independence at follow-up while the control group showed a signicant decrease in independence	Participants in the intervention group reported that assistance with daily activities was less essential than individuals in the control group at follow-up; However, there were no improvements in independence in daily living, mobility or a reduction in the number of occasions individuals were assisted with activities
Baron et al. [37]	127	22.4 on the Guys Neurological Disability Scale; patients with insomnia	N/R	48.1	telephone administered cognitive behavioral therapy	16 weeks	telephone administered supportive emotion-focused therapy	Improvements in depression and anxiety	Improvement in insomnia
Tompkins et al. [38]	3623	N/R		48.9 RM; 43.5 Control	PREP for participant and partner in workshop sessions or teleconference series; 8 hrs programming (1 or 2 days or 4–6 wks for teleconference)	In person 1–2 days or teleconference 4–6 weeks	No intervention	RM improvement with increased QoL at 3 months	Number of MS symptoms at baseline not signfiicantly different at baseline between groups but comorbidiities did (with control at fewer), controled at analysis stage. Improved communications; willingness to try; better prepared for issues; acquisition of tools to address MS issues with partner
Khan et al. [39]	101	EDSS between 2 and 8; KFS 0-2	10.69 (TR); 9.73 (Control)	49.5 TR; 51.1 Control	Individualised rehabilitation programme	12 months	waiting-list	MSIS and GHQ-28 assessed participation and QoL; no differences between control and treatment on MSIS physical or psychological or GHQ subscales	FIM motor scores improvement at statistically significant levels for 2 groups.
Sutherland et al. [40]	22	EDSS < = 5.0; no prior CB techniques for 6 months prior to study	Diagnosis : 9.36 yrs (TR); 6.45 yrs (Control)		AT program supervised training	10 weeks	No intervention	HRQOL positively affected;participants in relaxation less limited by physical findings but not for the AT . AT group positively impacted regarding role limitations due to emotional problems.	Pain dimension large effect of MSQOL indicates AT practice may associate with diminished pain perception.; Improved vigor (POMS); decreased perception of fatigue
Maguire [41]	33	N/R	N/R	45.13	Relaxation training and ongoing work with biologically oriented imagery.	6 days	Standard care	Imagery group subjects demonstrated significant reductions in state anxiety and significant alteration in their illness imagery	No significant differences were found between the two groups with regard to decrease in MS symptoms across time
Mathiowetz et al. [42]	169	Multiple Sclerosis Functional Composite score: −.97	15 years	48,8	Energy Conservation course	6 weeks	Waiting-list	increase self-efficacy and some aspects of quality of life	significant effects on reducing the physical and social subscales of Fatigue Impact Scale and on increasing the Vitality subscale of the SF-36 scores
Grossman et al. [43]	150	EDSS =3	8.7 years	47.29	A modified version of the Mindfulness-Based Stress Reduction (MBSR)	8 weeks	Usual Care	improvement on Quality of Life and other measures of well-being, for at least 8 months	Improvement on fatigue
Tavee et al. [44]	17	3,25 (Experimental group); 2,79 (controls)	10,4 (Experiemental group); 19,4 (Controls)	48,7	Meditation	2 months	Standard care	General improvement on mental health	Improvements on pain perception, phisical health, fatigue and vitality
Van Kessel et al. [45]	72	EDSS =3,45	6 years	45	CBT based on a cognitive behavior model of fatigue	8 weeks	relaxation training	A significant time effect was obtained for depression, anxiety and perceived stress, with both groups. CBT performed better, on this regard, at the post-treatment, but not at follow-up evaluations	Both CBT and RT appear to be clinically effective treatments for fatigue in MS patients, although the effects for CBT are greater than those for RT.
Mohr et al. [46]	121	EDSS =3,1	7,05 since diagnosis	42.66	individual stress management program	20–24 weeks	Waiting-list	Participants in the experiemental group reported lower level of distress	Reduction of brain lesions in comparison with the control group (lower number of new gadolinium-enhancing brain lesions on MRI)
Mohr et al. [47]	60	N/R	8.5 years	44,6	individual cognitive behavioral therapy, group psychotherapy	16 weeks	sertraline	Reductions on depression for each group	treatment for depression is associated with reductions in the severity of fatigue symptoms, and that this relationship is due primarily to treatment related changes in mood
Schwartz [48]	132	EDSS =4,7	7,9	43	coping skills group	8 weeks	peer telephone support	coping skills intervention yielded gains in psychosocial role performance, coping behavior, and numerous aspects of well-being. In contrast, the peer support intervention increased external health locus of control but did not influence psychosocial role performance or well-being	No differences between the two groups on physical limitations and fatigue
Wassem & Dudley [49]	27	EDSS =3,36	3,49	44	nursing intervention in promoting adjustment and symptom management	4 weeks	Not specified	Treatment participants had significant improvements in symptom management at the 4-yearfollow up	significant improvements in sleep and fatigue levels
Lincon et al. [50]	240				The assessment group received a detailed cognitive assessment; the treatment group received the same cognitive assessment and a treatment programme designed to help reduce the impact of their cognitive problems		No intervention	no effect of the interventions on mood, quality of life, subjective cognitive impairment or independence.	No differences among the three groups on perceived health
Mohr et al. [51]	14	EDSS =3,6	11.3	47.4	individual cognitive behavioral therapy, group psychotherapy	16 weeks	Sertraline	Reductions on depression for each group	successful treatment of MS depression (either pharmacologically or with psychotherapy) can reduce IFNg production by OKT3 or MBP-stimulated immune cells
Kopke et al. [52]	150	United Kingdom Neurological Disability Scale =7,9	5,2	38	Patient education program to enhance decision autonomy	4 hours	Standard care	The patient education program led to more autonomous decision making in patients with relapsing MS	The number of relapses reported by subjects in the experimental group was considerably lower than the one from controls

Overall, a total of 5,705 subjects with MS were included in the analysis, with a large study that included 3,623 subjects [38]. Setting aside that study, sample sizes ranged from 14 to 240 subjects. Most of these studies included people with MS with a limited physical disability (e.g., EDSS < 5.5) and with the average disease duration of 8 years and a mean age over 40 years. Articles that were included describe different psychological interventions for people with MS including cognitive-behavioral interventions, relaxation training, meditation, and stress management and coping skill promotion. There was variability about the duration of the intervention ranging from a week to two years, with an average length of two months. Control groups were composed primarily of subjects on a waiting-list or by no additional treatment group (usual care only). Four studies referred to a comparison between interventions, with controls receiving what was characterized as a less efficacious treatment or a gold-standard comparison.

Psychological variables were primary outcomes in all the included papers. The impact of the interventions on these outcomes was generally positive. Overall, psychological treatments produced an improvement in quality of life and psychological well-being, reducing depressive symptoms, anxiety and perceived stress. Most of the psychological treatments obtained positive effects. These effects were emphasized when the comparison was between the treatment and a usual care or a waiting-list control group.

The majority of the psychological effects on the physical symptoms were assessed using self-report measures, referencing the perception of physical variables or symptoms (e.g., fatigue, pain), or the perception of general physical health. Following the psychological intervention, perceptions of general health improved, with higher scores on the physical subscales on quality of life questionnaires. One symptom positively affected by psychological treatments is fatigue, in which subjects from experimental groups often reported a significant decrease in fatigue along with a subsequent reduction in physical limitations related to tiredness. Similarly, improvements in sleep disturbances, physical vitality, and vigor were reported. Psychological interventions also appeared to reduce the perception of pain.

Changes in physical issues do not result only from self-reported questionnaires but few studies investigated these changes with objective measurements. Results indicate that a stress-management intervention reduces the number of brain lesions associated with the relapsing-remitting process of MS, with a consequent reduction of crisis [46]. A short patient education program successfully reduced the number of relapses, compared to controls [52]. Furthermore, successful treatment of depression (either with psychological or pharmacological interventions) resulted associated with a reduction in non-specific and antigen-specific interferon production [51].

In general, with the caveat of the limited number of studies involved, when the psychological intervention lead to a better psychological outcome, such as the reduction of depressive symptoms or the improvement in psychological well-being, the assessed physical outcomes were positively influenced. A correlation can be observed between the extent of changes from a psychological perspective and the size of change in MS symptoms. More intense and efficacious psychological interventions lead to higher changes on a physical level than less intense behavioral treatments.

Articles included in the review seldom formally assessed the level of disability making it impossible to deeply investigate this aspect of findings. Future studies would benefit from a greater focus on assessment of disability in terms of functioning and inclusion of more non-self-report measures pre and post-intervention.

## Discussion

Results from the studies considered in this review suggest that psychological interventions may well have a positive effect on MS symptoms. In particular, fatigue, physical vitality, sleep disturbances and pain are the physical variables investigated that appeared to benefit from such interventions, together with perception of general health. Physical changes following a psychological intervention are reported on both self-report measurements and, in a more limited number of studies, on biological measures. If results are limited to questionnaire outcomes, it could be argued that psychological interventions may not provide objective changes, but could change the perception of the physical symptomology. It appears highly likely that both subjective and objective outcomes are moving toward one specific direction: that the mind does influence the body, even effecting MS symptoms. These findings should not surprise us, as we consider increasing evidence of the mind’s influence on the body. The idea that psychological treatments, however, may influence the physical expression (i.e., symptoms) of the disease itself is relatively new and few studies dare to explore this idea. In fact, relatively few papers considered some physical symptoms as a possible outcome for psychological interventions. Most of these studies only included a limited self-report assessment of health. It is possible that researchers in the MS field have thus far not tried to influence the possibility that interventions at the psychological level can result in positive effects on the body. Those who assessed physical changes with objective measures [46,51,52] found interesting results that surely deserve to be deepened and further explored. Since a cure for the various form of MS is not yet available, it makes sense to explore every possible therapeutic option, including the possibility that psychological treatment need not be palliative or burden-relief in nature [25,28]. Not surprisingly, fatigue, physical vitality and sleep disorder are often part of diagnostic criteria in the framework of depressive disorders. This is consistent with our hypothesis as an example of the effects that the mental domain can express over the body.

A few limitations of the present study should be noted. The majority of the studies included in the review utilized self-report measures for acquiring physical outcome data. As self-report measures are inherently based on subjective perception, the quality of their construct validity may have confounded the results of the review. Another limitation was that none of the studies reviewed included outcome data of any form of disability, possibly narrowing the scope of our assessment of physical outcomes. Finally, few studies in total were eligible for review; the studies included may therefore not adequately represent the general MS population.

## Conclusions

This brief review investigates the hypothesis that psychological interventions for individuals with multiple sclerosis have a positive impact not only at the psychological level, but also on the physical domains, in particular on symptoms of disease. Despite a paucity of studies that included assessment of physical variables as outcomes for psychological interventions, available data strongly suggest that the hypothesized connection does exist. In particular, fatigue, pain, physical vitality and quality of sleep, assessed by subjects’ evaluations, improve significantly after most of the interventions. Furthermore, a few cutting edge studies that assessed physical outcomes with objective measurements suggest that there are actual physical benefits, for example in terms of interferon level and brain lesions.

Our results indicate that there is a strong unexplored potential for psychological interventions to improve the quality of life of people with MS from both a psychological perspective and in terms of a reduction in symptoms. Given the potential improvement of well-being, we strongly urge research efforts be applied in this direction.

## References

[1] Switankowsky I (2000). Dualism and its importance for medicine. Theor Med Bioeth.

[2] Engel GL (1977). The need for a new medical model: a challenge for biomedicine. Science.

[3] Smith RC (2002). The biopsychosocial revolution. J Gen Intern Med.

[4] Fava GA, Belaise C, Sonino N (2010). Psychosomatic medicine is a comprehensive field, not a synonym for consultation liaison psychiatry. Curr Psychiatry Rep.

[5] Todaro JF, Shen B-J, Niaura R, Spiro Iii A, Ward KD (2003). Effect of negative emotions on frequency of coronary heart disease (the normative aging study). Am J Cardiol.

[6] Ho RC, Neo LF, Chua AN, Cheak AA, Mak A (2010). Research on psychoneu- roimmunology: does stress influence immunity and cause coronary artery disease?. Ann Acad Med Singapore.

[7] McBeth J, Macfarlane GJ, Silman AJ (2002). Does chronic pain predict future psychological distress?. Pain.

[8] Holgate ST, Komaroff AL, Mangan D, Wessely S (2011). Chronic fatigue syndrome: understanding a complex illness. Nat Rev Neurosci.

[9] Pressman SD, Cohen S (2005). Does positive affect influence health?. Psychol Bull.

[10] Langer EJ (1989). Mindfulness.

[11] Langer EJ (2009). Counter Clockwise: Mindful Health and the Power of Possibility.

[12] Rumrill PD (2009). Multiple sclerosis: medical and psychosocial aspects, etiology, incidence, and prevalence. J Vocational Rehabil.

[13] Fraser RT, Kraft GH, Ehde DM, Johnson KL (2006). The MS Workbook: Living Fully with Multiple Sclerosis.

[14] Isaksson AK, Gunnarsson LG, Ahlstrom G (2007). The presence and meaning of chronic sorrow in patients with multiple sclerosis. J Clin Nurs.

[15] Mullins LL, Cote MP, Fuemmeler BF, Jean VM, Beatty WW, Paul RH (2001). Illness intrusiveness, uncertainty, and distress in individuals with multiple sclerosis. Rehabil Psychol.

[16] Nosek MA, Hughes RB (2001). Psychospiritual aspects of sense of self in women with physical disabilities. J Rehabil.

[17] Ploughman M, Austin MW, Murdoch M, Kearney A, Godwin M, Stefanelli M (2012). The path to self-management: a qualitative study involving older people with multiple sclerosis. Physiother Can.

[18] Demirkiran M, Sarica Y, Uguz S, Yerdelen D, Aslan K (2006). Multiple sclerosis patients with and without sexual dysfunction: are there any differences?. Mult Scler.

[19] Çelik DB, Poyraz EC, Bingöl A, Idiman E, Özakbaş S, Kaya D: **Sexual dysfunction ın multiple sclerosis: Gender differences.** *Journal of the Neurological Sciences* in press. 10.1016/j.jns.2012.08.019 23079605

[20] Siegert RJ, Abernethy DA (2005). Depression in multiple sclerosis: a review. J Neurol Neurosurg Psychiatry.

[21] Kessler RC, McGonagle KA, Zhao S, Nelson CB, Hughes M, Eshleman S, Wittchen HU, Kendler KS (1994). Lifetime and 12-month prevalence of DSM-III-R psychiatric disorders in the United States. Results from the national comorbidity survey. Arch Gen Psychiatry.

[22] Gay MC, Vrignaud P, Garitte C, Meunier C (2010). Predictors of depression in multiple sclerosis patients. Acta Neurol Scand.

[23] Feinstein A (2011). Multiple sclerosis and depression. Mult Scler.

[24] Riether AM (1999). Anxiety in patients with multiple sclerosis. Semin Clin Neuropsychiatry.

[25] Fischer A, Heesen C, Gold SM (2011). Biological outcome measurements for behavioral interventions in multiple sclerosis. Ther Adv Neurol Disord.

[26] Zorzon M, Zivadinov R, Nasuelli D, Ukmar M, Bratina A, Tommasi M, Mucelli R, Brnabic‐Razmilic O, Grop A, Bonfigli L (2002). Depressive symptoms and MRI changes in multiple sclerosis. Eur J Neurol.

[27] Mohr DC, Hart SL, Julian L, Cox D, Pelletier D (2004). Association between stressful life events and exacerbation in multiple sclerosis: a meta-analysis. BMJ.

[28] Thomas PW1, Thomas S, Hillier C, Galvin K, Baker R (2006). Psychological interventions for multiple sclerosis. Cochrane Database Syst Rev.

[29] Moher D, Cook DJ, Eastwood S, Olkin I, Rennie D, Stroup DF (1999). Improving the quality of reports of meta-analyses of randomised controlled trials: the QUOROM statement. Lancet.

[30] Shekelle PG, Woolf SH, Eccles M, Grimshaw J (1999). Clinical guidelines: developing guidelines. Br Med J.

[31] Barlow J, Turner A, Edwards R, Gilchrist M (2009). A randomised controlled trial of lay-led self-management for people with multiple sclerosis. Patient Educ Couns.

[32] Stuifbergen AK, Becker H, Blozis S, Timmerman G, Kullberg V (2003). A randomized clinical trial of a wellness intervention for women with multiple sclerosis. Arch Phys Med Rehabil.

[33] Ghafari S, Ahmadi F, Nabavi M, Anoshirvan K, Memarian R, Rafatbakhsh M (2009). Effectiveness of applying progressive muscle relaxation technique on quality of life of patients with multiple sclerosis. J Clin Nurs.

[34] Tesar N, Baumhackl U, Kopp M, Günther V (2003). Effects of psychological group therapy in patients with multiple sclerosis. Acta Neurol Scand.

[35] Forman AC, Lincoln NB (2010). Evaluation of an adjustment group for people with multiple sclerosis: a pilot randomized controlled trial. Clin Rehabil.

[36] O’Hara L, Cadbury H, De Souza L, Ide L (2002). Evaluation of the effectiveness of professionally guided self-care for people with multiple sclerosis living in the community: a randomized controlled trial. Clin Rehabil.

[37] Baron KG, Corden M, Jin L, Mohr DC (2011). Impact of psychotherapy on insomnia symptoms in patients with depression and multiple sclerosis. J Behav Med.

[38] Tompkins SA, Roeder JA, Thomas JJ, Koch KK (2013). Effectiveness of a relationship enrichment program for couples living with multiple sclerosis. Int J MS Care.

[39] Khan F, Pallant JF, Brand C, Kilpatrick TJ (2008). Effectiveness of rehabilitation intervention in persons with multiple sclerosis: a randomised controlled trial. J Neurol Neurosurg Psychiatry.

[40] Sutherland G, Andersen MB, Morris T (2005). Relaxation and health-related quality of life in multiple sclerosis: the example of autogenic training. J Behav Med.

[41] Maguire BL (1996). The effects of imagery on attitudes and moods in multiple sclerosis patients. Altern Ther Health Med..

[42] Mathiowetz VG, Finlayson ML, Matuska KM, Chen HY, Luo P (2005). Randomized controlled trial of an energy conservation course for persons with multiple sclerosis. Mult Scler.

[43] Grossman P, Kappos L, Gensicke H, D’Souza M, Mohr DC, Penner IK, Steiner C (2010). MS quality of life, depression, and fatigue improve after mindfulness training A randomized trial. Neurology.

[44] Tavee J, Rensel M, Pope Planchon S, Stone L (2010). Effects of meditation on pain and quality of life in multiple sclerosis and polyneuropathy: a controlled study. Neurology.

[45] van Kessel K, Moss-Morris R, Willoughby E, Chalder T, Johnson MH, Robinson E (2008). A randomized controlled trial of cognitive behavior therapy for multiple sclerosis fatigue. Psychosom Med.

[46] Mohr DC, Lovera J, Brown T, Cohen B, Neylan T, Henry R, Siddique J, Jin L, Daikh D, Pelletier D (2012). A randomized trial of stress management for the prevention of new brain lesions in MS. Neurology.

[47] Mohr DC, Hart SL, Goldberg A (2003). Effects of treatment for depression on fatigue in multiple sclerosis. Psychosom Med.

[48] Schwartz CE (1999). Teaching coping skills enhances quality of life more than peer support: results of a randomized trial with multiple sclerosis patients. Health Psychol.

[49] Wassem R, Dudley W (2003). Symptom management and adjustment of patients with multiple sclerosis a 4-year longitudinal intervention study. Clin Nurs Res.

[50] Lincoln NB, Dent A, Harding J, Weyman N, Nicholl C, Blumhardt LD, Playford ED (2002). Evaluation of cognitive assessment and cognitive intervention for people with multiple sclerosis. J Neurol Neurosurg Psychiatry.

[51] Mohr DC, Goodkin DE, Islar J, Hauser SL, Genain CP (2001). Treatment of depression is associated with suppression of nonspecific and antigen-specific TH1 responses in multiple sclerosis. Arch Neurol.

[52] Köpke S, Kasper J, Mühlhauser I, Nübling M, Heesen C (2009). Patient education program to enhance decision autonomy in multiple sclerosis relapse management: a randomized-controlled trial. Mult Scler.

